# Potential Protection of *Coeloglossum viride* var. Bracteatum Extract against Oxidative Stress in Rat Cortical Neurons

**DOI:** 10.1155/2013/326570

**Published:** 2013-12-15

**Authors:** Zhe Guo, Rui-Yuan Pan, Xiao-Yan Qin

**Affiliations:** ^1^Beijing Engineering Research Center of Food Environment and Health, Minzu University of China, Beijing 100081, China; ^2^College of Life & Environmental Science, Minzu University of China, Beijing 100081, China; ^3^Emergency Department, The Third Affiliated Hospital of Guangxi Medical University, Nanning 530021, China

## Abstract

The present study explored the neuroprotective effect of *Coeloglossum viride* var. bracteatum extract (CE) against oxidative stress in rat cortical neurons. The results demonstrated that administration of CE inhibited hydrogen peroxide-induced neurotoxicity tested by MTT, LDH release, and TUNEL assays. We further found that CE inhibited the activation of caspase-3 (Csp3) induced by hydrogen peroxide. Moreover, CE was found to reverse the hydrogen peroxide-induced downregulation of active AKT and Bcl-2. We then showed that the neuroprotective effect of CE was blocked by adding the AKT inhibitor, Ly294002. Thus, our data strongly indicated that CE played a neuroprotective role against oxidative stress-induced neurotoxicity.

## 1. Introduction

*Coeloglossum viride* var. bracteatum extract (CE) is extracted from a plant called *Coeloglossum viride* var. bracteatum, an orchidaceae family plant. CE is widely used as a traditional Chinese medicine in the Northwest of China, especially in Tibet, Qinghai, Gansu Inner Mongolia, and Shanxi provinces [1]. It has been described as a traditional Chinese medicine; CE increases body fluid production and vital energy is beneficiary to memory and tranquilization [1]. In 2004, CE has been identified as a mixture of four compounds [2]. More recently, CE has been shown to rescue learning and memory deficit induced by scopolamine in rodents [3].

Alzheimer's Disease (AD) is a progressive neurodegenerative disease characterized by extracellular senile plaques composed of amyloid beta and intracellular neurofibrillary tangles (NFTs) [4]. Neuronal loss in AD is thought to be contributed by amyloid beta toxicity [5], and growing evidence suggests that the amyloid beta toxicity is mediated by oxidative damage in neurons [4]. Numerous studies suggested that oxidative stress plays a major role in the pathogenesis of AD. It has been observed that oxidative stress is increased in brains from patients with AD [6]. Moreover, reduction of antioxidant enzyme activity including superoxide dismutase (SOD) and catalase has been reported in brains from patients with AD [7, 8]. In addition, deficiency of superoxide dismutase D1 (Sod1) in a mouse model of AD resulted in accelerated amyloid beta plaque formation and memory deficit, and the phenomena were mediated by oxidative damage [9]. Interestingly, a recent study found that an antioxidant MitoQ blocked increased oxidative stress, increased amyloid beta plaque formation, and elevated caspase activity and synaptic loss in an animal model of AD [10]. Thus oxidative stress is a promising therapeutic target for treating AD.

In our present study, we investigated the role and mechanism of action of CE in neuroprotection against oxidative stress. Our results for the first time suggested that CE might activate AKT signaling pathway to upregulate the expression of the antiapoptotic protein Bcl-2 to mediate neuroprotection during stress.

## 2. Material and Methods

### 2.1. Primary Cortical Neuronal Culture and Treatments

The cortical neuronal cultures were prepared as described previously. Tissues were dissected from the whole brain tissues of newborn SD rats (Experimental Animal Center of Peking University Health Science Center, Beijing, China) in Dulbecco's modified Eagle's medium (DMEM) (Invitrogen). The tissues were dissociated mechanically and then incubated with 0.25% trypsin (Invitrogen) for 30 minutes at 37°C. The mixture was then filtered through a nylon mesh to obtain homogenous suspension. After filtering the mixture through 70 *μ*m sterilized filters, the flow-through was centrifuged to pellet cells. Cells were then resuspended in DMEM with 10% fetal bovine serum (FBS), 2 g/L HEPES, penicillin G (100 U/mL), and 100 ug/mL streptomycin (all from Invitrogen). Cells were plated in poly-L-lysine-coated plates or coverslips and maintained in a humidified incubator with 5% CO_2_ and 95% O_2_ at 37°C. 10 *μ*M cytosine arabinoside (Sigma) was supplemented after plating for 2–4 days to inhibit glia cell growth. Cells were treated after 7-8 days in culture. CE (a gift from Dr. Li Tang, Department of Pharmacology, Minzu University of China) and AKT inhibitor Ly294002 (cell signaling) were added freshly into culture medium during treatments.

### 2.2. Measurement of Cell Death: MTT Assay, LDH Release Assay, and TUNNEL Assay

The viability of cells after various treatments was estimated by their ability to reduce the dye methyl thiazolyl tetrazolium (MTT, Sigma) to blue formazan crystal. Cells cultured in 96-well plate for 7 days were gently washed with 0.01 M PBS. After wash, 90 *μ*L of medium with 10 *μ*L of 5 mg/mL MTT solution was added to each well and the plate was maintained at 37°C for 2–4 hours. Then the reactions were dissolved in DMSO for quantification by measuring the absorption at 570 nm using a microplate spectrophotometer (Bio-Rad), representing relative cell viability.

The cytotoxicity of cells after various treatments was evaluated by lactate dehydrogenase (LDH) release. This was achieved with a CytoTox 96 Non-Radioactive Cytotoxicity Assay kit according to the manufacturer's instructions (Promega).

Cell death was examined after treatments by fixing cells in fresh 4% paraformaldehyde and 4% sucrose in PBS for 20 minutes at room temperature and permeabilized in 0.1% triton X-100 and 0.1% sodium citrate in PBS for 2 minutes on ice. Terminal deoxynucleotidyl transferase-mediated dUTP nick-end labeling (TUNEL) staining was performed using the *in situ* cell death detection kit I as described by the manufacturer (Roche). The coverslips were then washed once in distilled water for 5 minutes and mounted on glass slides to be observed under a fluorescence microscope. The percentage of cell death was determined by the ratio of the number of TUNEL-positive cells over the total of 100 cells in one count. The average of 5 counts was calculated as the percentage of neuronal cell death in a certain treatment.

### 2.3. Western Blots

The neurons cultured in 6-well plates were washed three times with 0.01 M PBS; 100 *μ*L of cell lysis buffer with 1% phenylmethanesulfonyl fluoride (PMSF) was added into each well and cells were harvested with cell scrapers. The extracts were iced for 30 minutes and centrifuged at 14,800 g for 15 minutes, and the supernatant was harvested. Denatured protein samples diluted with loading buffer were loaded equally to each lane and separated by 10% SDS-PAGE and then blotted onto a polyvinylidene fluoride (PVDF, Millipore) membrane. The membrane was then incubated for 1 hour in blocking buffer (tris-buffered saline containing 5% no-fat milk powder) at room temperature. The membrane was incubated at 4°C with the primary antibodies, washed with trisbuffered saline Tween-20 (TBST), and incubated again with horseradish peroxidase (HRP)-conjugated secondary antibodies (Jackson ImmunoResearch Laboratories) followed by washing. The primary antibodies used include purified polyclonal anti-*β*-actin antibody (Santa Cruz), monoclonal antiactivated Csp3 antibody (cell signaling) and polyclonal anti-Bcl-2 antibody (Santa Cruz), and monoclonal anti-p-AKT antibody (cell signaling). Immunoblots were developed in the presence of enhanced chemiluminescence reagents, and the images detected in X-ray films were quantified by densitometric scanning using Gel Imaging Analysis System Gel-Pro 4400 (Media Cybernetics).

### 2.4. Statistical Evaluation

All data are presented as means ± S.E.M. Statistical significance (**P < 0.01 or ***P < 0.001) among groups was determined by two-tailed Student's *t*-test.

## 3. Results

**Figure 1:**
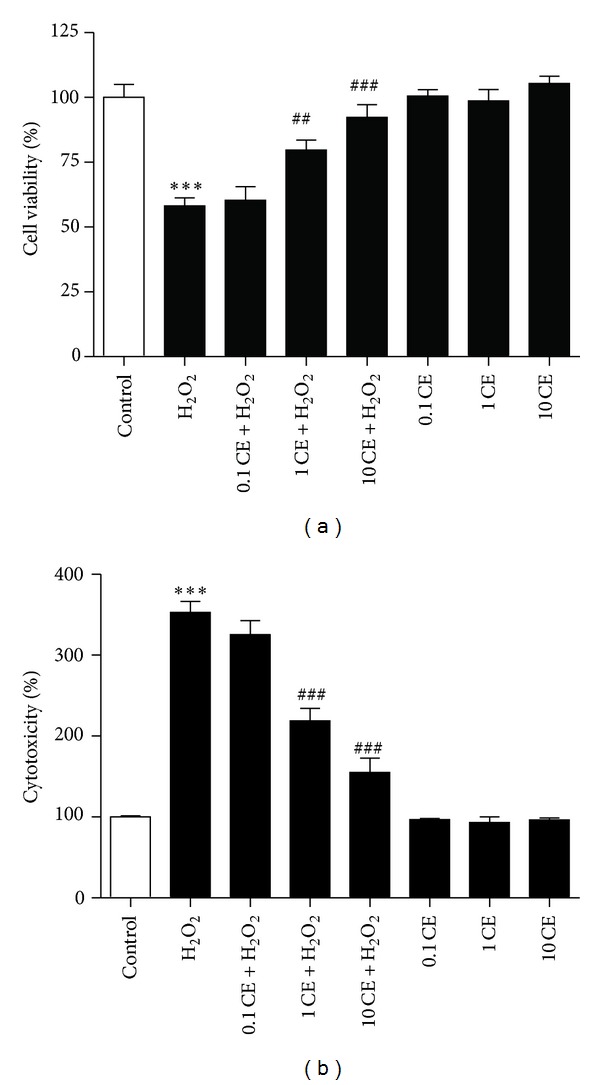
Protective effects of CE against oxidative stress in primary cultured cortical neurons. (a) Bar graphs showing the reduced cell viability after H_2_O_2_ treatment that was significantly attenuated by the treatment of the neurons with 1 mg/mL and 10 mg/mL CE as tested by MTT assay. (b) Bar graphs showing the H_2_O_2_ induced cytotoxicity that was significantly reduced by the treatment of the neurons with 1 mg/mL and 10 mg/mL CE as tested by LDH release assay. ∗ refers to a comparison of the control group and # refers to a comparison of the H_2_O_2_ group.

To explore the neuroprotective effect of CE in primary cultured cortical neurons, we treated the cortical neurons with vehicle, 0.1 mg/mL CE, 1 mg/mL CE, or 10 mg/mL CE in the absence or presence of 100 *μ*M H_2_O_2_. As shown in Figure 1(a), the cell viability of cultured cortical neurons significantly decreased after 24 h incubation with H_2_O_2_ compared to the control group as tested by MTT assay; however, 1 mg/mL CE or 10 mg/mL CE significantly inhibited the H_2_O_2_ induced decrease of cell viability, while CE alone had no marked influence on the cell viability in primary cultured cortical neurons (Figure 1(a)).

To confirm the neuroprotective effect of CE against oxidative stress, LDH release assay was used to measure the cytotoxicity. Results showed that H_2_O_2_ significantly increased the cytotoxicity in the neurons compared to the control group; however, treatment with 1 mg/mL CE or 10 mg/mL CE significantly reduced the cytotoxicity induced by H_2_O_2_, while CE alone had no marked influence on the cytotoxicity in the neurons (Figure 1(b)).

**Figure 2:**
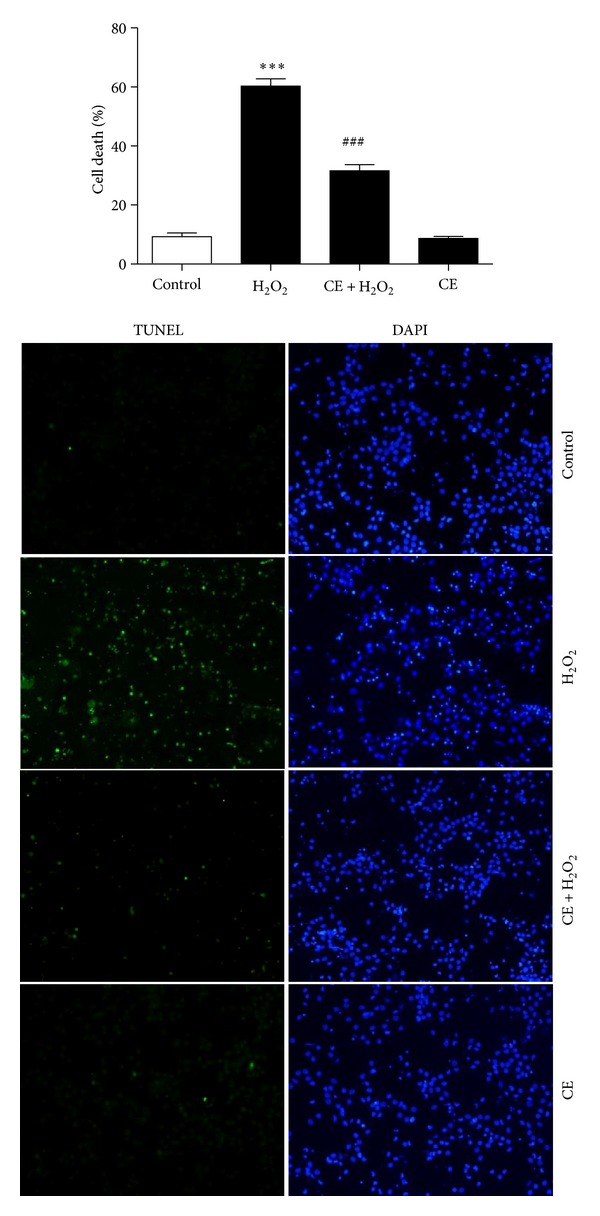
Photomicrographs and histograms showing that treatment with CE reduced H_2_O_2_ induced cell death assessed by TUNEL assay. ∗ refers to a comparison of the control group and # refers to a comparison of the H_2_O_2_ group.

Since the above results suggested that the neuroprotective effect of CE peaks at 10 mg/mL CE, this concentration was used in all subsequent experiments. Results from TUNEL assay further confirmed the neuroprotective effect of CE in the primary cultured cortical neurons. As shown in Figure 2, the number of died cortical neurons significantly increased after treatment with H_2_O_2_ compared to the control group; the number of died cells significantly reduced by incubation of the neurons with CE. Moreover, CE alone had no marked influence on the cell death of the neurons.

**Figure 3:**
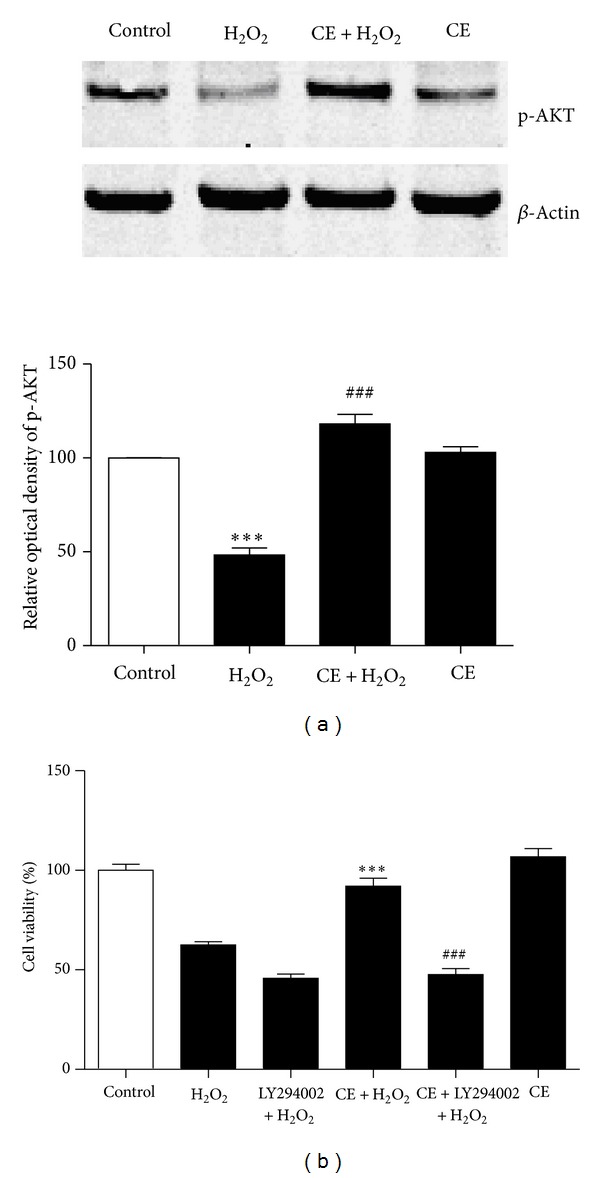
AKT signaling pathway is required for the neuroprotective effect of CE. (a) representative western blot probed with an antibody specific against p-AKT after various treatments in the neurons. Actin served as an internal control. Summary of the optical density of p-AKT. Results showed that CE significantly inhibited H_2_O_2_ induced decreases in the content of p-AKT. ∗ refers to a comparison of the control group; # refers to a comparison of the H_2_O_2_ group. (b) Bar graphs showing that the neuroprotective effect of CE was blocked by AKT inhibitor Ly294002, suggesting that AKT signaling pathway mediated the neuroprotective effect of CE in primary cultured cortical neurons.

**Figure 4:**
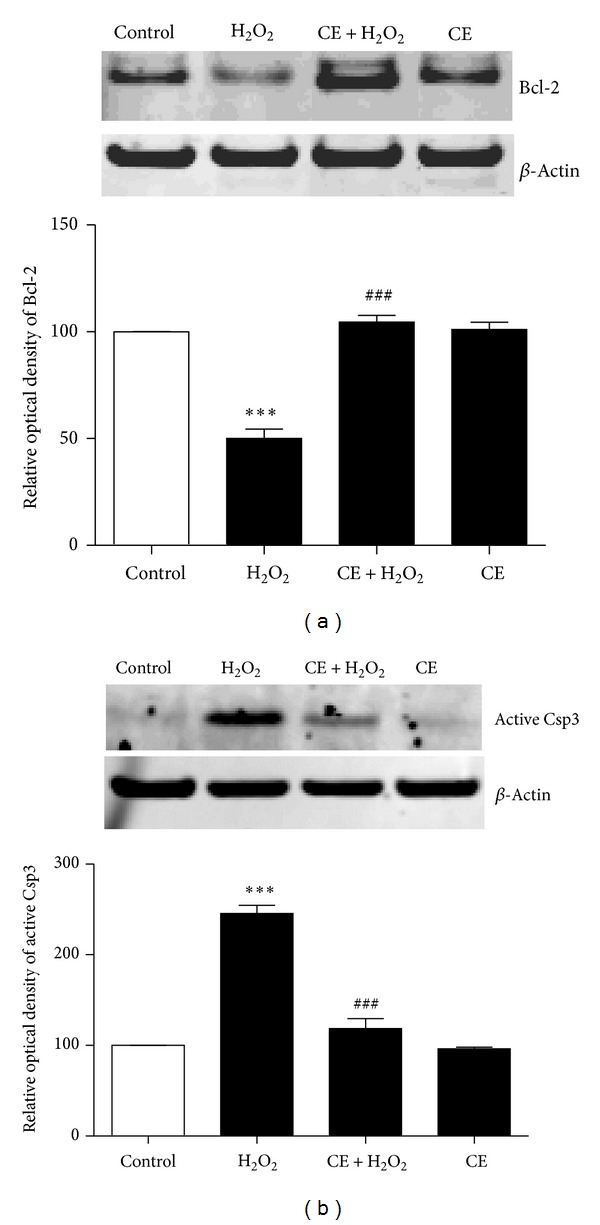
Neuroprotection by CE involves Bcl-2 and Csp3. (a) Representative western blot probed with an antibody specific against Bcl-2 after various treatments in the neurons. *β*-Actin served as an internal control. Summary of the optical density of Bcl-2. Results showed that CE significantly inhibited H_2_O_2_ induced decreases in the content of Bcl-2. (b) Representative western blot probed with an antibody specific against active Csp3 after Csp3 various treatments in the neurons. Actin served as an internal control. Summary of the optical density of active Csp3. Results showed that H_2_O_2_-induced activation of Csp3 was blocked by CE. ∗ refers to a comparison of the control group; # refers to a comparison of the H_2_O_2_ group.

To investigate the mechanism underlying the neuroprotective effect of CE, we tested the expression of apoptotic or antiapoptotic proteins kinase B (AKT), Bcl-2, and Csp3 after different treatments. Results from western blot showed that the phosphorylation of AKT was decreased significantly after H_2_O_2_ treatment compared to the control group; however, incubation of the neurons with CE reversed the downregulation of phosphorylated AKT induced by H_2_O_2_ (Figure 3(a)). Moreover, as shown in Figure 4(a), the antiapoptotic protein Bcl-2 was significantly decreased in the cortical neurons treated with H_2_O_2_ compared to the control group; however, incubation with CE significantly inhibited the H_2_O_2_ induced decrease of Bcl-2 in the neurons compared to the H_2_O_2_ treated group. Furthermore, we found that the H_2_O_2_ induced activation of Csp3 was blocked by incubation with CE.

To confirm the regulation of the AKT required for the neuroprotective effect of CE, we use the AKT inhibitor, Ly294002. Western blot showed that the phosphorylation of AKT was abolished after incubation with Ly294002 in the neurons (data not shown), demonstrating the specificity of the inhibitor. Next, the primary cultured cortical neurons were treated with vehicle or 10 *μ*M Ly294002 for 30 min. Subsequently, H_2_O_2_ was added to the neurons in the absence or presence of CE for 24 h. As shown in Figure 3(a), Ly294002 completely blocked the neuroprotective effect of CE against oxidative stress in the cortical neurons.

## 4. Discussion

Neurodegenerative diseases are a wide class of hereditary and sporadic conditions characterized by progressive nervous system dysfunction. These disorders include Alzheimer's disease (AD), Parkinson's disease (PD), Huntington's disease (HD), and prion diseases which are caused by a combination of genetic and environmental factors. With more than 200 billion dollars for studying and treating AD alone globally per year, neurodegenerative diseases remain without curative or preventive treatments, and the available therapies provide only symptom improvements. Recently, evidence is emerging that natural products are promising targets to treat neurodegenerative diseases such as AD and PD with less side effects. For example, studies have demonstrated that curcumin and green tea polyphenols protected against amyloid beta-induced neurotoxicity *in vitro* [11–13]. Moreover, curcumin suppressed oxidative damage and blocked cognitive deficits in an animal model of AD [14]. Here we demonstrated that CE protected against hydrogen peroxide-induced cell death in primary cultured neurons. This is consistent with our previous findings that CE inhibited amyloid beta-induced neurotoxicity. Moreover, CE rescued ischemia-induced neuronal death and cognitive impairment in rats [15]. Thus our data support the hypothesis that oxidative stress plays an important role in mediating amyloid beta-induced neuropathology, memory deficits, and the progression of AD.

AKT signaling pathway is a major pathway for survival and neuroprotection [16]. It has been reported that the p-AKT levels are decreased in brains from patients with AD and an animal model of AD [17, 18]. Here we showed that oxidative stress decreased the level of phosphorylated AKT and CE reversed the oxidative stress-induced downregulation of active AKT, which has antiapoptotic property. More importantly, we found that AKT inhibitor blocked the neuroprotective effect of CE against oxidative stress in primary cultured cortical neurons, suggesting the neuroprotective effect of CE was mediated by AKT signaling pathway. Hence our results are consistent with a role for AKT signaling pathway in stress and the progression of AD.

Bcl-2 family is a large family of proteins with pro- or antiapoptotic properties. These include Bax which is a proapoptotic protein and Bcl-2 which is an antiapoptotic protein. Upon apoptosis, Bax undergoes a conformational change which exposes its hydrophobic C terminal; then it is oligomerized and translocated to mitochondria from cytosol to form pores, which then leads to the release of cytochrome c and the activation of caspase, eventually leading to cell death [19, 20]. In contrast, Bcl-2 interacts with Bax to prevent the pore formation in mitochondria to mediate survival and neuroprotection [21]. Given the important role of Bcl-2 (Bax) to caspase signaling pathway in apoptosis, it is not surprising to find that the expression of Bcl-2 was altered in brains from patients with AD [22]. Furthermore, Csp3 has been observed to be increased and enriched in postsynaptic densities in AD [23]. Interestingly, a study found that the inhibition of Csp3 activity rescued the synaptic failure and cognitive dysfunction in an animal model of AD [24]. Here we demonstrated that the decrease in Bcl-2 protein caused by oxidative stress was prevented by treatment with CE. Moreover, our results showed that the activation of Csp3 caused by oxidative stress was blocked by treatment with CE. Based on our new findings, CE could be a potential therapeutic target for AD. Thus continued investigation into the function of CE is warranted.
